# Comprehensive Analysis of High-Sensitive Flow Cytometry and Molecular Mensurable Residual Disease in Philadelphia Chromosome-Positive Acute Leukemia

**DOI:** 10.3390/ijms26052116

**Published:** 2025-02-27

**Authors:** Ana Paula de Azambuja, Ana Lucia Vieira Mion, Yara Carolina Schluga, Miriam Perlingeiro Beltrame, Alexandra Cristina Senegaglia, Vaneuza Araujo Moreira Funke, Carmem Bonfim, Ricardo Pasquini

**Affiliations:** 1 Hospital de Clínicas, Universidade Federal do Paraná, Curitiba 80060-900, Brazil; ana.azambuja@hc.ufpr.br (A.P.d.A.);; 2 Duke Children’s Hospital, Durham, NC 27710, USA

**Keywords:** acute lymphoblastic leukemia, Philadelphia chromosome-positive acute leukemia, measurable residual disease, flow cytometry, chronic myeloid leukemia blast phase, leukemia associated-immunophenotype

## Abstract

Monitoring measurable residual disease (MRD) is critical for the management of B-cell acute lymphoblastic leukemia (B-ALL). While a quantitative assessment of BCR::ABL1 transcripts is standard for Philadelphia chromosome-positive cases (Ph+ ALL), a multiparameter flow cytometry (FCM) is commonly used for MRD detection in other genetic subtypes. A total of 106 B-ALL patients underwent genetic and phenotypic analyses. Among them, 27 patients (20 adults and 7 children) harbored the *t(9;22)(q34.1;q11.2)* translocation and/or the BCR::ABL1 rearrangement. A high correlation between the BCR::ABL1 transcript levels (PCR-MRD) and a standardized FCM-based method for MRD detection (FCM-MRD) was observed (*r* = 0.7801, *p* < 0.001), with a concordance rate of 88% (κ = 0.761). The FCM detected MRD in 82.9% of the samples with transcript levels of > 0.01%. The CD34+CD38−/dim blast pattern was significantly more frequent in Ph+ ALL (77.7%), compared to other B-ALL cases (20.2%, *p* < 0.0001). Additionally, Ph+ ALL exhibited a higher expression of CD66c+/CD73+ (94.0% vs. 56.9%), CD66c+/CD304+ (58.8% vs. 6.9%), and CD73+/CD304+ (75.5% vs. 15.5%) than the other B-ALL subtypes (*p* < 0.001). In conclusion, this high-sensitivity FCM-MRD demonstrated comparable performance to the PCR-MRD, serving as a complementary tool for MRD assessment in Ph+ ALL. Moreover, a distinct leukemia-associated immunophenotype was identified, highlighting potential biomarkers for MRD monitoring.

## 1. Introduction

The recurrent chromosomal and molecular abnormalities that characterize acute lymphoblastic leukemia (ALL) subtypes are of great value for diagnosis, risk stratification, disease monitoring, and treatment selection [1,2]. Among these, the *BCR::ABL1*-positive B-ALL, here called Philadelphia chromosome-positive acute leukemia (Ph+ ALL), occurs in 2–5% of pediatric and 15–25% of adult ALL cases [3]. This genetic abnormality is pivotal in determining the prognostic stratification and guiding the therapeutic decisions in acute leukemia. The incorporation of an intensive chemotherapy combined with tyrosine kinase inhibitors (TKIs) has transformed the treatment landscape for Ph+ ALL, with 94–100% of patients achieving complete hematological remission with minimal induction-related mortality [3,4].

Philadelphia chromosome-positive diseases demonstrate significant variability in their immunophenotype, genetic, and molecular features [5]. The distinction between “de novo” Ph+ ALL and chronic myeloid leukemia presenting as lymphoid blast phase (CML-BP) remains a clinical challenge, particularly in patients without a prior diagnosis of CML [5,6]. The isoform of the BCR::ABL1 fusion transcript appears to reflect the distinct biological origins and clinical implications of these conditions [7,8,9], since the resulting oncoproteins (p190, p210, or p230) differ significantly in their characteristics, kinase activity, and the signaling pathways they activate [10,11,12]. However, these entities cannot be clearly distinguished by the immunophenotype or by the differences in the fusion protein. Otherwise, the underlying difference seems to reflect the target cell for the transformation event, with either a multipotent or a later progenitor serving as the target [13].

In the recent years, the assessment of measurable residual disease, formerly minimal residual disease (MRD), has become a cornerstone in managing acute leukemia, including the Philadelphia chromosome-positive B-ALL. MRD monitoring provides a more refined evaluation of the treatment response [14,15], which is particularly critical in patients undergoing hematopoietic stem cell transplantation (SCT), where the absence of a detectable disease before the transplant is strongly associated with improved long-term outcomes [16,17].

Immunoglobulin rearrangement testing (Ig/TCR) is the standard method for the MRD assessment in ALL, but the technical expertise and instrumentation required limit its application to specialized centers [18]. MRD is typically monitored in patients with Ph+ ALL using a BCR::ABL1 transcript quantification through a specific real-time quantitative polymerase chain reaction (RQ-PCR) assay [19,20]. PCR-MRD provides a specific and highly sensitive blast detection, allowing for a timely intervention and adjustment of therapy [20]. However, recent studies have reported that BCR::ABL1 rearrangements can persist in non-lymphoblastic cells in patients with negative Ig/TCR results. These discrepancies have been attributed to multilineage involvement and clonal hematopoiesis [12,13].

Although a quantitative measurement of the fusion gene effectively tracks transcript levels in Ph+ B-ALL cases, in patients with other genetic subtypes, multiparameter flow cytometry (FCM) profiling has been used for MRD detection with a good performance [21,22]. In fact, FCM-MRD detection represents a technically challenging and rapidly advancing application of flow cytometry. The earlier studies, which primarily employed three- or four-color techniques, faced significant challenges in distinguishing the leukemic cells from normal B-lineage subpopulations in bone marrow regeneration, both during and after treatment [22,23]. Furthermore, the accuracy and reliability of conventional FCM are influenced by several critical factors, including the number of acquired cellular events and parameters analyzed, as well as the specificity of studied antigens [24,25].

Over the past decade, technological advancements have significantly improved FCM-MRD detection, with six- to ten-color FCM methods demonstrating substantial enhancements in sensitivity and precision [25]. The validation of a flow cytometry for high-sensitive testing has expanded its usefulness by incorporating new markers and sample preparation techniques that allow for acquiring more cellular events. In this way, several groups have been introducing 8 to 12-color FCM-MRD methods in B-cell precursor acute lymphoblastic leukemia (B-ALL), the sensitivity of which could be compared with current molecular techniques [24,26,27,28].

In 2017, the EuroFlow™ group proposed a sensitive and reliable flow cytometry method for assessing MRD, where the leukocyte markers, corresponding antibodies and fluorochromes were strategically selected using a multidimensional principal component analysis (PCA) based on their contribution to distinguishing between abnormal and regenerating precursor B-cells [24]. The inclusion of markers associated with genetic abnormalities, such as CD66c, CD73 and CD304, has significantly enhanced the detection of residual blasts [29]. By combining these markers in a single fluorescence channel, this protocol identified abnormal expression patterns in around 70% of B-ALL patients [24]. Additionally, a total lysis protocol, combined with standardized instrument settings, ensures a consistently high analytical sensitivity [30].

This study focuses on Philadelphia chromosome-positive acute leukemia patients, examining the correlation between BCR::ABL1 transcript levels (PCR-MRD) and a standardized flow cytometry-based MRD detection method (FCM-MRD) in B-ALL. Our objective was to validate a high-sensitivity FCM protocol for application in both Ph+ and non-Ph cases. This approach is particularly relevant in our setting, where implementing MRD assessments using Ig/TCR qPCR or next-generation sequencing (NGS) presents significant challenges. While FCM-MRD requires specialized equipment and trained personnel, it is often more accessible in resource-limited settings, compared to NGS or Ig/TCR qPCR, due to its lower cost and faster turnaround time. Additionally, we comprehensively analyzed the clinical, phenotypic, and genetic-molecular characteristics of a unique cohort of Philadelphia chromosome-positive acute leukemia patients, offering valuable insights into this high-risk group.

## 2. Results

### 2.1. Patient Cohort

A retrospective cohort of 125 acute lymphoblastic leukemia (ALL) patients were followed between 2017 and 2024 in the Flow Cytometry Laboratory of Hospital de Clínicas da Universidade Federal do Paraná, Curitiba, Brazil. Cases of T-cell ALL (*n* = 18) and mixed-phenotype acute leukemia (MPAL) My/T (*n* = 1) were excluded from this study.

A total of 106 B-ALL patients were available for genetic and phenotypic analysis. Among them, 27 patients (20 adults and 7 children) with the t(9;22)(q34.1;q11.2) translocation and/or BCR::ABL1 rearrangement were selected for a detailed analysis of transcript subtypes and clinical, morphological, and phenotypic characteristics.

The control group included 79 B-ALL patients (27 adults and 52 children) with availability for a high-sensitivity MRD analysis. This group comprised 24 cases with diploid karyotypes and 36 with cytogenetic abnormalities: 12 hyperdiploidy karyotypes, 5 *ETV6::RUNX1* cases, 2 *E2A::PBX* cases, and 10 with *KMT2A* rearrangements (*KMT2Ar*). Seven patients exhibited varied abnormalities, including two cases with del6, isochromosomes i(9) and i(7), one add7, one t(8;12) and one del(14). Additionally, nineteen patients lacked metaphases or molecular results.

**Figure 1:**
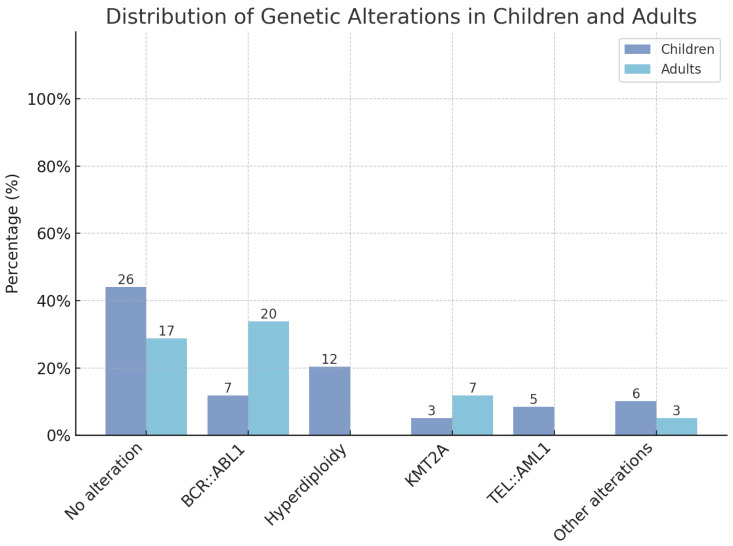
Distribution of available recurrent genetic alterations between children and adult cohorts.

Figure 1 shows the percentage distribution of genetic alterations and differences between children and adult cohorts.

### 2.2. Cytogenetic and Molecular Biology

A cytogenetic analysis and a molecular screening at diagnosis revealed nine patients with additional cytogenetic abnormalities, including complex/variant Philadelphia chromosomes, and five cases with cryptic Philadelphia chromosomes harboring BCR::ABL1 rearrangements. The B/My MPAL patient had a hypodiploid karyotype with a rare (7;14) translocation (see Supplementary Table S1).

Regarding BCR::ABL1 fusion transcript types, 17 patients (63.0%) expressed only the p190 (e1a2) fusion and were classified as having “de novo” Ph+ ALL, while 10 patients (37.0%) expressed the p210 fusion and were categorized as having secondary lymphoid blast transformations of chronic myeloid leukemia (CML-BP).

Among CML-BP patients, three (10.0%) expressed only the p210 transcript: two with e13a2 (b2a2) and one with e14a2 (b3a2). Seven cases (23.3%) co-expressed p190 and p210 transcripts, including two with e14a2 (b3a2) and e1a2, three with e13a2 (b2a2) and e1a2, and two with three transcripts: e13a2 (b2a2), e14a2 (b3a2), and e1a2.

Supplementary Table S1 summarizes the genetic and molecular characteristics of the 27 Philadelphia chromosome-positive patients.

### 2.3. Clinical Characteristics and Outcomes of BCR::ABL1-Positive B-ALL Group

The *BCR::ABL1*-positive B-ALL group included 7 children and 20 adults, with a median age of 31.3 years (range 3.8–75.6). Nine patients presented leukocytosis and neutrophilia on the initial complete blood count, suggesting a multilineage involvement, although only two had documented chronic-phase CML with prior tyrosine kinase inhibitor treatment. The extramedullary disease was observed in four patients: two with central nervous system involvement, one with breast infiltration, and one with liver infiltration (detailed in Supplementary Table S2).

Patients received induction polychemotherapy combined with TKIs (imatinib, nilotinib, or dasatinib), per referral center protocols. Allogeneic stem cell transplantation (SCT) was performed for eligible patients in the first complete remission [31].

After induction chemotherapy, only two patients achieved negative FCM-MRD and PCR-MRD status. Fifteen had positive MRD levels (>0.01%), while eight had active disease (MRD > 5%). Two patients exhibited primary refractory disease and did not receive further therapy. Early mortality occurred in five older adults (18.5%) due to induction failure or refractory disease. Three pediatric and two young adult (AYA) patients achieved durable remission with TKIs and high-dose chemotherapy without SCT. Six out of nineteen patients undergoing SCT experienced relapses, with four requiring a second SCT. Four relapsed patients and six with transplant-related toxicity died. Nine patients remained in extended follow-up post-SCT.

The mean age at diagnosis was comparable between the *BCR::ABL1*-positive B-ALL subgroups: 29.3 years for p190 Ph+ ALL patients and 36.3 years for p210 CML-BP patients. Initial blood counts revealed significantly lower mean leukocyte levels in the Ph+ ALL group compared to the CML-BP group (17,358/μL vs. 129,711/μL, *p* = 0.01). No significant differences were found between the groups regarding precursor left shift, blastic predominance, hemoglobin levels, or platelet counts. The overall survival (OS) was 62.4% for the “de novo” Ph+ ALL group and 56.2% for the CML-BP group (HR 0.701, 95% CI 0.2098–2.226, *p* = 0.53). Event-free survival and relapse rates were comparable between “de novo” Ph+ ALL and CML-BP groups. As no other notable differences were identified, the patients were analyzed collectively as a single Philadelphia chromosome-positive B-ALL group (Ph+ ALL).

### 2.4. Immunophenotypic Analysis

#### 2.4.1. Maturation Profile

In this series, Ph+ ALL patients predominantly exhibited a typical common pre-B cell immunophenotype (CD19 and CD10 positive, cytoplasmic IgM-negative), like other B-ALL cases. However, four cases displayed weak or heterogeneous CD10 expression (see Supplementary Table S3).

A CD34 strong expression was observed in nearly all Ph+ ALL cases (96.3%), compared to 48.8% of other B-ALL patients. Conversely, CD38 expression was negative or dim in most Ph+ patients (22.2% CD38 positive), versus the 62% in other B-ALL cases. As a result, the CD34++CD38-/dim blast pattern was significantly more frequent in Ph+ ALL patients (77.7%) than in other B-ALL cases (20.2%, *p* < 0.0001).

**Figure 2:**
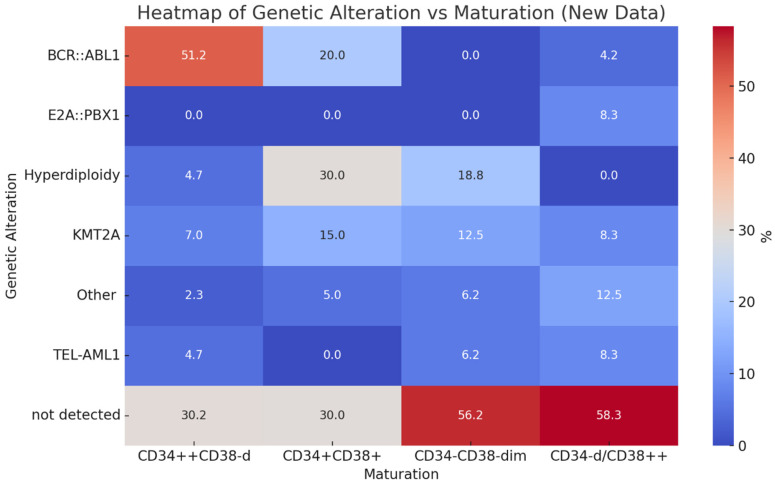
Heatmap illustrating the correlation between genetic alterations and maturation phenotypes, emphasizing the significant association of expression pattern CD34++CD38−/dim with *BCR::ABL1*-positive B-ALL cases and CD34+CD38+ with hyperdiploidy.

Figure 2 presents a heatmap illustrating the correlation between genetic alterations and maturation phenotypes, showing the strong association of the CD34++CD38-/dim expression pattern with *BCR::ABL1*-positive B-ALL cases.

#### 2.4.2. Leukemia-Associated Immunophenotype

Although *BCR::ABL1*-positive B-ALL did not correlate with any specific marker (*p* = 0.34), all cases in this series exhibited at least one leukemia-associated immunophenotype (LAIP) suitable for MRD monitoring via flow cytometry (Supplementary Table S2).

CD66c expression was evaluated in 20 patients (74%), with 14 (70%) showing CD66c positivity and 6 (30%) exhibiting negative expression. Markers CD73 and CD304 were assessed in 17 patients (63%). In this group, eight cases (47%) were double-positive for CD66c+/CD73+ with CD304 negative, and the other eight cases were triple-positive for CD66c+/CD73+/CD304+. Additionally, nine cases (53%) were double-positive for CD66c+/CD304+, with CD73− expression. CD123 was tested in 26 cases (93%) and was positive or dimly positive in 19 cases (73%).

Notably, the six patients with negative CD66c expression demonstrated strong positivity for CD73 and/or CD304, ensuring all Philadelphia chromosome-positive cases expressed antigens suitable for flow cytometric MRD monitoring.

**Figure 3:**
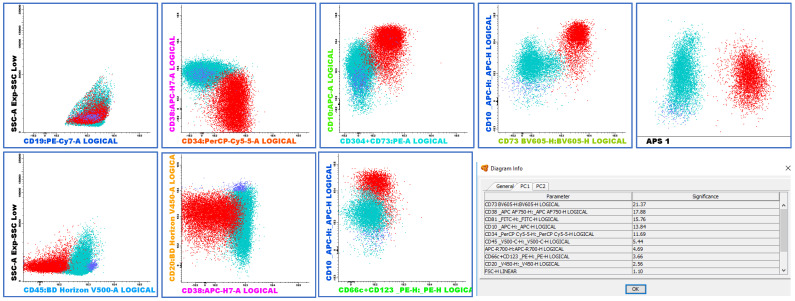
Representative MRD flow cytometry plots and APS1 view showing lymphoblasts (red) exhibiting the phenotypes CD10++, CD20 negative, CD34+, CD38-/dim, CD66c/CD123 negative and CD73/CD304++. Positivity of CD73 was confirmed on BV605 fluorochrome. APS view shows clear distinction between blasts (red), mature B-cells (blue), and B-cell precursors (green).

Among the cases tested for the three markers (CD66c, CD304, and CD73), eight of the seventeen Ph+ cases (47.0%) and twenty-five of the fifty-eight non-Ph+ cases (43.1%) were positive for all three markers, with no significant difference between the groups. Figure 3 illustrates a representative case with CD73+ and CD34+CD38−/dim lymphoblasts.

Table 1 shows the immunophenotypic differences observed between Philadelphia chromosome-positive cases and other genetic subtypes of B-ALL.

#### 2.4.3. Comparison Between Ph+ ALL and Other B-ALL Cases

In the B-ALL control cohort, normal karyotypes were positively associated with CD66c+ expression (*p* = 0.006), while other genetic abnormalities showed no significant association with specific LAIPs (*p* = 0.076).

Among the cases with confirmed genetic abnormalities, *TEL::AML1* was significantly associated with CD73+ and CD73+/CD304+ expression (*p* = 0.002); *E2A::PBX1* was linked to CD73+ expression (*p* < 0.001); and hyperdiploidy karyotypes correlated with positivity for two or three markers (CD66c+CD73+CD304+, *p* = 0.05). In contrast, *KMT2A* rearrangements were associated with the loss of markers, including CD10 (Pro-B ALL phenotype), and were negatively associated with CD66c, CD73, and CD304 positivity.

Double positivity for CD66c+/CD73+ (56.9% vs. 94.0%), CD66c+/CD304+ (6.9% vs. 58.8%), and CD73+/CD304+ (15.5% vs. 75.5%) was significantly lower in non-Ph+ B-ALL patients compared to *BCR::ABL1*-positive B-ALL patients (*p* < 0.001).

**Figure 4:**
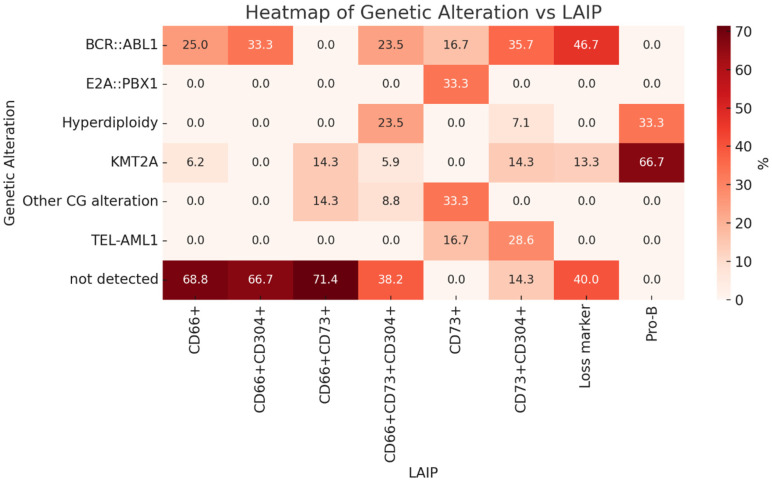
Heatmap illustrating the correlation between genetic alterations and LAIP phenotypes, showing possible association between CD66c+ and CD304+ with *BCR::ABL1* cases.

Figure 4 illustrates the correlation between genetic alterations and leukemia-associated immunophenotypes using a heatmap.

### 2.5. Correlation Between FCM-MRD and PCR-MRD in Philadelphia Chromosome Positive Cases

A total of 19 diagnostic and 90 follow-up samples were analyzed simultaneously using RQ-PCR and flow cytometry (see Supplementary Table S4). Of the 109 samples studied, RQ-PCR and FCM provided concordant qualitative results in 91 cases, resulting in concordant results in 88% of cases (Kappa = 0.761, *p* < 0.001).

**Figure 5:**
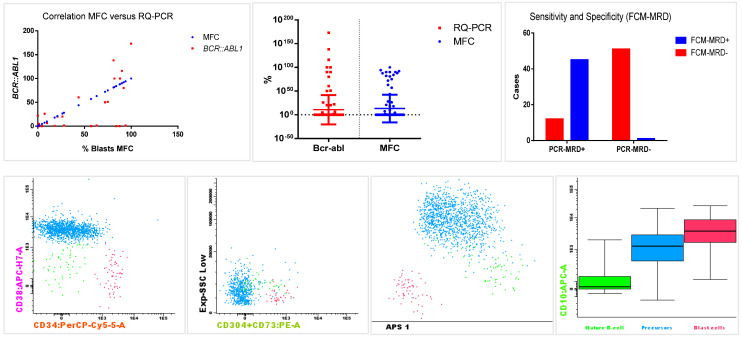
Scatter plots showing a correlation between the percentage of blasts detected by multiparameter flow cytometry (MFC) and BCR::ABL1 transcript. Histogram showing the number of discrepant cases between PCR-MRD and FCM-MRD. Flow cytometry plots and APS view from reanalysis of discrepant FCM-MRD-/PCR-MRD+ case, showing 70 cellular events CD34+, CD38dim, CD304/CD73+, CD10++, considered as blasts (pink); mature (green) and precursor B-cells (blue) are well distinguished by the APS1 view.

The quantitative comparison revealed a significant correlation between the percentage of blasts detected by FCM-MRD and the number of transcripts (*BCR::ABL1/ABL* ratio) measured by RQ-PCR [31]. The Pearson correlation (r = 0.7801; 95% CI: 0.69–0.84, *p* < 0.001) and the Spearman correlation (r = 0.8618; 95% CI: 0.802–0.9045, *p* < 0.001) were both statistically significant, as shown in Figure 5.

#### 2.5.1. FCM-MRD Sensitivity Compared to PCR-MRD

Flow cytometry exhibited a sensitivity of 78.9% (95% CI: 66.1–88.6%) and specificity of 98.8% (95% CI: 89.7–99.9%) for blast detection compared to RQ-PCR transcript analysis. Likewise, the positive predictive value was 97.8% (95% CI: 88.5–99.9%), and the negative predictive value reached 80.9% (95% CI: 69.1–89.7%).

Among the 52 PCR-MRD negative samples, 51 (98.1%) were also FCM-MRD negative. For MRD-positive samples, FCM showed a detection rate of 78.9% (45 out of 57) compared to RQ-PCR. In the cases with >0.01% positivity PCR-MRD, FCM reliably detected ALL cells in 82.9% (39 out of 47) of the samples.

Considering the discordant results, 12 were PCR-MRD positive but FCM-MRD negative, and in one sample MRD was detected by a flow cytometry but not by RQ-PCR. The samples tested positive when using RQ-PCR, and the transcript level was detected at very low expression levels, with MRD indices ranging from 0.007 to 0.105 (median 0.085).

#### 2.5.2. Discrepant Cases

After reviewing the discrepant cases, three samples with no detectable blasts in the highly sensitive flow cytometry test (10,000,000 events acquired), RQ-PCR detected the e14a2 transcript, but it was not quantified, at levels below the limit of detection (LoD < 0.0001%). In one of these samples, the flow cytometry revision detected 0.0007% of blasts (70 cellular events in 10,000,000) that were not accounted for in the initial FCM-MRD report (see Figure 5).

The other eight discordant samples had insufficient cellular events in FCM to meet the LoD and LLoQ thresholds for detecting MRD below 0.01%. Eight of nine samples were collected during the first year of the project, before the full introduction of bulk lysis and standardized FCM-MRD protocol. The discrepant sample, in which FCM found 0.07% leukemic blasts and PCR-MRD was negative, was subsequently FCM-MRD negative in the two sequential reports.

The discrepant cases are detailed in Table 2.

#### 2.5.3. MRD Kinetic Analysis of Selected Cases

Most cases with the p190 (e1a2) transcript achieved PCR-MRD negativity following treatment or bone marrow transplantation. In patients with multiple BCR::ABL1 transcript types, the p190 (e1a2) transcript is typically cleared before the p210 isoform post-treatment. Among the six cases analyzed, p190 was cleared first in five, while in one case, p210 disappeared before p190.

Supplementary Figure S1 shows box plots revealing the distribution of each MRD time point for groups Ph+ and Ph negative ALL. The plots show overlaps and differences in their spreads and central tendencies, but there is no significance between the groups (d15 *p* = 0.273; d33 = 0.099; d90 = 0.483).

Supplementary Tables S5 and S6 show two examples of the dynamic of MRD disappearance in both transplant and non-transplant settings.

## 3. Discussion

### 3.1. Correlation Between FCR-MRD and PCR-MRD–Sensitivity

Measurable residual disease in Philadelphia chromosome-positive disease, achieved with the *BCR::ABL1* quantitative method, is the standard of care in CML and Ph+ acute leukemias [3,15]. However, assessing the MRD using a multiparametric flow cytometry has been the recommended practice in patients with other genetic subtypes [24,26,28]. The choice of methods usually depends on the expertise, resources, and design of the clinical trial established in each setting.

This study observed a good correlation and concordant qualitative results between blast percentages detected by a high-sensitive flow cytometry and BCR::ABL1 transcript levels in 109 concomitant samples. These findings are consistent with previous studies that emphasize the potential complementary roles of molecular and flow-based assays [16,21]. Flow cytometry showed high sensitivity and specificity, detecting MRD in 82.9% of cases with >0.01% residual disease by RQ-PCR, confirming its utility for clinical monitoring. Notably, discordances primarily involved cases with very low transcript levels (<0.001%) or insufficient cellular events for FCM thresholds.

A detailed review of FCM data revealed missed blasts in one sample, emphasizing the importance of meticulous analysis and expertise to ensure accurate results. Furthermore, the FCM-MRD samples with low sensitivity were from the project’s first year, before the full introduction of bulk analysis and complete protocol in our center. This underscores the importance of optimizing sample quality, rigorous quality control, and even the level of training experts in flow cytometry analysis [32].

Our results align with the recent studies, demonstrating a good concordance between the flow cytometry and molecular MRD detection techniques in Ph+ and other B-ALL [23]. Singh et al. compared a single 10-color tube with the measurement of MRD by Ig/TCR qPCR in Philadelphia-negative disease and *BCR::ABL1* (fusion gene) RQ-PCR in Ph+ disease and reported discordance in 13 samples, including one case where the flow cytometry detected an impending relapse, missed by molecular methods. Among the samples in which MRD was not detected by flow cytometry, there was only one case in which *BCR::ABL1* was detected at 0.065%. Similarly to our results, the BCR::ABL1 level was <0.01% in the nine discordant samples, demonstrating an adequate sensitivity and a good correlation between the techniques [23].

These results support the idea that FCM-MRD and PCR-MRD produce similar results and may be useful in monitoring disease treatment. Moreover, combining two independent methods, PCR-MRD and FCM-MRD, minimizes the risk of false-negative results, and a positive result from either assay usually indicates MRD [21,22,23].

### 3.2. Philadelphia Chrmosome-Positive Acute Leukemia Differences

The outcomes among the Philadelphia chromosome-positive acute leukemia patients are quite heterogeneous, and accurately differentiating “de novo” Ph+ ALL from CML-BP remains a significant challenge [5,7,8,9]. The long isoform of the BCR::ABL1 (p210) fusion, predominantly associated with the CML-like background, appears to originate from hematopoietic stem cells, and typically results from e13a2 (b2a2) or e14a2 (b3a2) fusion transcripts. Conversely, the short isoform (p190), arising from a minor BCR rearrangement producing the e1a2 fusion transcript, is mainly associated with Ph+ ALL and likely originates in a B-cell progenitor [5,6,13]. These distinctions may reflect potential differences in the biological origin of the diseases and significantly impact clinical management [11,13,33].

In our study, 63% of patients with acute-phase disease expressed the p190 transcript (typical Ph+ ALL), while 37.0% expressed the p210 transcript, including seven cases with co-expression of two or three transcripts. Despite the limited number of patients, our results align with a pivotal German study, which reported that 77% of Ph+ ALL cases expressed the p190 transcript, 20% the p210 transcript, and 3% co-expressed both [6]. Furthermore, the p210 expression was associated with high-risk features, such as older age, elevated white blood cell counts [7,9], and persistence of the transcript despite the absence of detectable lymphoblasts, compared to those with the p190 transcript [7,8,9]. Notably, we identified seven cases with multiple transcript types and the p190 (e1a2) isoform generally disappeared earlier than the p210 isoform following treatment. These findings highlight the dynamic nature of MRD kinetics and underscore the importance of combining PCR-MRD and FCM-MRD for comprehensive monitoring and management of Ph+ disease.

The standard treatment for Ph+ ALL combines chemotherapy with TKI therapy, achieving remission rates of 90–100%. This is typically followed by allogeneic hematopoietic stem cell transplantation, which offers curative potential in approximately two-thirds of cases [1]. Achieving a complete molecular remission before transplant is critical for long-term outcomes, underscoring the importance of MRD monitoring to guide therapy and detect potential resistance or relapse [16,17].

The introduction of immunotherapy, including anti-CD19 and anti-CD22 therapies, has further emphasized the need to differentiate typical Ph+ ALL from CML-like cases to optimize treatment strategies in future protocols [33,34]. Recent studies suggest that while the SCT remains the preferred approach for CML-like ALL, treatment of typical Ph+ ALL is evolving, with a growing recommendation for TKI-based regimens combined with risk-adapted chemotherapy or immunotherapy [11].

### 3.3. Leukemia-Associated Immunophenotype and Genetic Correlation

Detectable MRD after induction therapy in B-ALL is commonly associated with cytogenetic aberrations linked to poor outcomes, such as *BCR::ABL1* and *KMT2A* rearrangements [2]. Furthermore, the phenotypic portrait of B-ALL is marked by great complexity and heterogeneity of profiles, reflected by the expression of LAIP markers [29].

The widespread use of flow cytometry to investigate MRD in acute leukemia has motivated researchers to identify membrane markers with a specificity that can be associated with the most common cytogenetic alterations [35]. For example, CD66c {carcinoembryonic antigen-related cell adhesion molecule} and CD123 {alpha-chain of the interleukin-3 receptor} expression has been correlated, although not specifically, with *BCR::ABL1*-positive B-ALL and hyperdiploid cases [36,37]. Similarly, CD73 {ecto-5′-nucleotidase} and CD304/neuropilin-1 are potentially associated with *ETV6-RUNX1* rearrangements [38,39,40], and inversely associated with *TCF3-PBX1* fusion gene, whereas the antibody 7.1 {chondroitin sulfate proteoglycan, NG2} is a well-established marker correlated to 11q23/*KMT2A* rearrangements [35].

By applying the MRD search panel, suggested by EuroFlow™, with four differential markers and the new analysis tools (APS view and reference image), we identified at least one LAIP in each Ph+ ALL patient. This has greatly improved the discrimination of hematogones from pathological B cells in these cases. Our results were similar to recent studies where CD304 positivity was associated with the presence of the *BCR::ABL1* gene [39,40]. This study suggests that the positivity for CD73, CD304, and CD66c antigens may serve as an independent and reliable marker for MRD detection in Ph+ ALL [36,39,40]. This combination of markers has proven to be a valuable tool for distinguishing leukemic cells from normal hematopoietic cells.

### 3.4. Difference from Normal (Dif-N) Strategy and Maturation Pattern

The different-from-normal (Dif-N) strategy distinguishes normal B-cell precursors (hematogones/BCP) from B lymphoblasts based on maturation profiles. This can be challenging because normal BCP shares many immunophenotypic characteristics with B lymphoblasts. A perfect knowledge of normal immunophenotypic patterns associated with normal B-cell maturation and normal marrow regeneration is required [41].

An interesting study of deep molecular profiling revealed three transcriptomic subtypes of *BCR::ABL1* lymphoblastic leukemia, each representing a maturation arrest at a stage of B-cell progenitor differentiation. An earlier arrest was associated with lineage promiscuity, treatment refractoriness, and poor patient outcomes. Later arrests were associated with lineage fidelity, durable leukemia remissions, and improved patient outcomes. Each maturation arrest was marked by specific genomic events controlling different B-cell development transition points [13].

We found no significant differences in maturation pattern, with a similar frequency of CD10+ common type ALL in Ph+ cases compared to those in other genetic subtype groups. However, a notable observation was the significantly lower expression of CD38 and higher expression of CD34 at the surface of B lymphoblasts with t(9;22) in comparison with B lymphoblasts without other recurrent cytogenetic alteration.

Although we found no association between the BCR::ABL1 fusion type with clinical or phenotypic factors, the high prevalence of the CD34+CD38-/dim pattern in CD19+CD10+ lymphoblasts was surprising. This pattern is rare in bone marrow, particularly when these cells co-express specific immunophenotypes associated with leukemia, such as CD73, CD304 and/or CD66c [24,35].

This finding parallels the CD34+CD38−/dim leukemic stem cell (LSC) phenotype observed in myeloproliferative neoplasms, since these LSCs are believed to initiate leukemia and play a pivotal role in disease relapse [42]. Notably, in chronic myeloid leukemia, a unique surface expression profile has been identified in LSCs, characterized by consistent expression of markers such as CD25+, CD26+, CD56+, and IL-1RAP+ [43]. In contrast, while the CD34+CD38−/dim phenotype with aberrant marker expression (e.g., CD45RA, CD123, or CD26) is well-documented in CML and acute myeloid leukemia, a definitive LSC phenotype for lymphoid leukemia remains undefined. This underscores the need for further research to establish specific phenotypic markers for LSCs in lymphoid leukemia.

### 3.5. Key Points

This study highlights the complexities of Philadelphia chromosome-positive acute leukemia and emphasizes the critical importance of integrating comprehensive molecular and phenotypic evaluations to improve prognostic accuracy and refine treatment strategies. Despite certain limitations, including its retrospective design, small sample size, and lack of standardization in chemotherapy protocol monitoring, this study demonstrates the feasibility of using this highly sensitive assay as an alternative method for MRD detection in both Ph+ and other B-ALL cases.

The consistent expression of key immunophenotypic markers may indicate fundamental biological differences in leukemic cells, suggesting phenotypic immaturity and a potential link to the aggressiveness and clinical behavior of Ph+ ALL. These findings support the continued integration of FCM-MRD into routine clinical practice, particularly in resource-limited settings where access to advanced molecular diagnostics remains limited [32,44].

## 4. Materials and Methods

### 4.1. Casuistic

A retrospective cohort of 125 acute lymphoblastic leukemia (ALL) patients were followed between 2017 and 2024 in the Flow Cytometry Laboratory of Hospital de Clínicas da Universidade Federal do Paraná, Curitiba, Brazil.

106 B-ALL patients were analyzed for genetic subtypes, clinical features, and phenotypic markers, at 25.5% Ph+ ALL (20 adults and 7 children). Samples performed simultaneously with FCM-MRD and quantitative PCR-MRD had concordance rates and discrepancies assessed.

The control cohort consisted of 79 B-cell ALL patients, 52 children and 27 adults, with normal karyotypes or genetic abnormalities other than Ph+. Cases of T-cell ALL and MPAL My/T were excluded from this study.

### 4.2. Diagnostic Procedures

Standard diagnostics were performed according to local practice [3]. Philadelphia chromosome-positive acute leukemia was defined by the presence of t(9;22)(q34;q11) translocation and the resulting BCR::ABL1 rearrangement [45], and clinical/outcome data were collected from the hospital information system.

Patients expressing the p190 (e1a2) transcript were classified as “de novo” Ph+ ALL. Patients expressing the p210 transcript were classified as having secondary lymphoid blast transformations of chronic myeloid leukemia (CML-BP). The acute phase was considered if more than 20% of lymphoblasts were in the bone marrow in morphology. Using standard techniques, a conventional chromosomal analysis was performed on G-banded metaphase cells, prepared from unstimulated bone marrow aspirate cultures. Twenty metaphases were analyzed, and the results were reported using the International System for Human Cytogenetic Nomenclature.

An immunophenotypic analysis defined subgroups of B-cell ALL as follows: pro-B ALL, TdT+ and CD19+, with negative CD10, cytoplasmatic (cy) IgM and surface (S) IgM; B-common ALL, TdT, CD19 and CD10 positive, with cyIgM and Sig negative; and pre-B common ALL, TdT, CD19 and CD10 positive, and negative cyIgM and SigM. A mixed phenotypic acute leukemia (MPAL) was defined as the presence of both myeloperoxidase and strong CD79a/CD19 (B/My) or cytoplasmatic myeloperoxidase and CD3 (T/My) in the same pathological cell.

A complete remission was characterized by a morphological remission, with fewer than 5% blasts in the bone marrow and no evidence of extramedullary disease. Central nervous system relapses were diagnosed when leukemic cells were detected in the cerebrospinal fluid.

### 4.3. Multiparameter Flow Cytometry

Bone marrow samples were studied during the acute phase diagnosis, and MRD analysis was performed at follow-up, according to the clinical and chemotherapy protocol used. To obtain more cells, bone marrow samples were processed following a bulk lysis protocol [30]. In brief, 2000  µL of whole bone marrow specimens were lysed with an ammonium chloride-based lysis solution and stained with monoclonal antibodies before a red cell lysis using FACS Lyse (Becton Dickinson, Franklin Lakes, NJ, USA). In diagnostic samples, 100,000 to 200,000 events per tube were collected, and up to 5,000,000 events per tube were used for MRD analyses. Samples were considered MRD-positive if at least 20 (limit of detection—LoD), and at least 50 clustered events were recorded (lower limit of quantitation—LLoQ). The LoD and LLoQ were determined for each case by considering the total cells analyzed in each tube.

Diagnostic antibody panels proposed by the EuroFlow™ group were used in diagnostic samples [46]. The B-ALL MRD panel comprised two 8-color tubes with seven backbone markers: CD81 FITC (clone JS81), CD34 PercpCy5.5 (clone 8G12), CD19 Pecy7 (clone J3-119), CD10 APC (clone MEM-78), CD20 V450 (clone L27), CD38 APC-H7 (clone HB7) and CD45 V500c (clone 2D1). To better discriminate the lymphoblasts from normal BCP, the antibodies CD66c PE (clone B62) and CD123 PE (clone 9F5) were put in the first tube, and CD73 PE (clone AD-2) and CD304 PE (clone Neuropilin-1) in the second tube^14^. Personalized panels followed patients with MPAL and T-cell precursor leukemia.

FACSCanto II™ (BD Biosciences, Franklin Lakes, NJ, USA) equipped with BD FACSDiva v9.0 Software (BD Biosciences, Erembodegem, Belgium) was used, and instrument settings were generated according to EuroFlow™ guidelines [46]. The FCS files from the sample databases were manually analyzed using Infinicyt™ software version 2.0 (Cytognos SL, Salamanca, Spain), and all cases were critically reviewed.

### 4.4. Strategy for Immunophenotypic Analysis

FCM-MRD tubes were analyzed using merge and automatic population separation (APS) strategies (software Infinicyt™, Cytognos). First, B cells were selected based on CD19 expression, excluding duplicates and debris identified by their forward (FSC) and side (SSC) light scattering characteristics. The B cell gate was selected in the side scatter versus CD19 and included B lymphoblasts, hematogones, or normal B lymphocytes.

A Boolean gating approach was applied to exclude plasma and stromal cells, using CD81 versus CD38 and CD81 versus CD73 dot plots, respectively. Subsequently, intersections between CD10 versus CD20, CD20 versus CD38, and CD34 versus CD38 were analyzed to delineate the maturation stages of B lymphoid precursors, categorizing them as pre-B-I, pre-B-II, and mature B cells.

**Figure 6:**
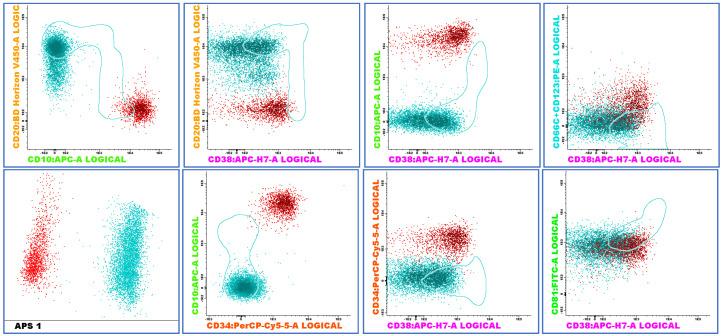
An illustrative example of a positive MRD case where maturation markers and the APS strategy successfully differentiate blasts (red) from residual normal mature B-lymphocytes in the sample (green). The figure shows blasts (red) exhibiting the phenotypes CD10+++, CD20−, CD34+, CD38−, CD81+, and weak CD45+. PCR-MRD analysis showed e13a2 and e1a2.

Normal leukocytes were considered internal control populations to determine the threshold of the positivity of each marker (each normal sub-population of leukocytes was selected depending on previously described profiles in the literature). A positive expression of each evaluated marker by populations of interest was defined when at least 20% of the population expressed the marker, with help from APS and reference image Infinicyt™ tools. This strategy is exemplified in Figure 6.

The aberrant B lymphoblasts were identified by comparing their immunophenotypic profiles to normal maturation patterns (different from normal strategy-DifN) and leukemia-associated immunophenotypes (LAIPs), focusing on phenotypic aberrations or abnormal expression levels of B-lineage markers. The presence of aberrant expression of the four PE-linked markers (CD123, CD66c, CD73, and CD304) in the B-cell population was analyzed using the statistical strategy PCA incorporated in Infinicyt software™ (APS view) to optimize the detection of abnormal lymphoblasts. The abnormal blast population was quantified as a percentage of total nucleated blood cells.

The presence of any level of abnormal cells in FCM-MRD constitutes the FCM-MRD-positive group (MRD+), whereas patients with less than 10^−4^ (<0.01%) blast cells were considered the FCM-MRD-negative (MRD−) group. Patients with more than 5% of blasts were considered to have active disease/relapse disease. The abnormal blast population was quantified as a percentage of total nucleated blood cells.

### 4.5. Molecular Biology Procedures

Levels of *BCR-ABL* fusion transcripts were quantified in a TaqMan™-based, real-time competitive PCR, using *ABL1* as a control gene [31,45,47]. This RT-PCR assay detected p190 (e1a2) and p210 (e13a2 and e14a2) transcripts simultaneously. The standard curve was based on serial dilutions of the plasmid linearized with the *BCR-ABL1* insert (pNC190/G) and (pNC210/G) [31]. The p210 transcripts were quantified in duplicate by Q-PCR (ABI PRISM 7500, Life Technologies, Carlsbad, CA, USA) using the TaqMan™ hydrolysis probe system G [47]. The results were reported as a percentage (%) of *BCR::ABL1* and *ABL1* copy numbers (*BCR::ABL1/ABL1* × 100).

The *BCR::ABL1/ABL1* ratio was multiplied by the conversion factor (CF) to present the values on an international scale (IS). The Molecular Biology Laboratory’s conversion factor is 0.51, which was determined by comparing the results of BCR::ABL1 transcript quantification in 30 samples, analyzed at CHC-UFPR and the reference laboratory at the Institute of Medical and Veterinary Science, Adelaide, Australia. The CF value was confirmed using a second set of 30 samples from the same reference laboratory. Samples were considered acceptable for analysis when ABL1 copies exceeded 32,000. A nested PCR was performed on all samples that did not have transcripts detected by competitive PCR to confirm the results.

### 4.6. Comparison of Monitoring Techniques

The samples analyzed in parallel using quantitative RQ-PCR (*BCR::ABL*/ABL ratio) and blast count found in flow cytometry were compared to validate the sensitivity for MRD monitoring. Cases were considered discordant if the flow cytometry did not identify any residual population of lymphoblasts, and if the BCR::ABL1 transcripts were detectable in at least two separate samples or vice versa. Discordances were recorded if the level of lymphoblasts identified in the flow was more than ten times lower than that predicted by the BCR::ABL1 transcript level, based on the reduction in baseline levels. Given the sensitivity of the RQ-PCR and FCM monitoring techniques, transcript levels more than 3 logs below the baseline level (approximately 1 in 1000 Ph+ cells or less) were not considered discordances [16,21].

### 4.7. Statistical Analysis

Descriptive statistics were utilized to summarize baseline patient characteristics. For group comparisons, *t*-tests were applied to normally distributed data, while non-parametric tests, such as the Mann–Whitney U test and Fisher’s exact test, were employed for non-normally distributed data or categorical variables.

A quantitative comparison using general associations of FCM and PCR data as continuous variables was evaluated using bivariate non-parametric Pearson and Spearman correlation statistics. A qualitative analysis of concordance between the FCM and PCR results and a binary classification test was performed. These tests evaluated the performance of FCM relative to PCR as the reference method, providing measures such as sensitivity (percentage of concordant positive cases relative to the total PCR-MRD positive cases), specificity (percentage of concordant negative cases relative to the total PCR-MRD negative cases), and overall concordance (percentage of all concordant cases).

The coefficient of determination and the Cohen’s Kappa (K) test were used to evaluate the data between groups. Two-tailed *p* values < 0.05 were considered statistically significant. Statistical comparisons were performed using GraphPad Prism version 9.0 (GraphPad Software, San Diego, CA, USA).

### 4.8. Ethics Committee

This study was approved by the CHC-UFPR Medical Ethics Committee under protocol number CAAE 84969718.0.000.0096 and conducted according to the principles of the Declaration of Helsinki. Medical records were accessed in the hospital’s database system. The patient or legal guardian consented to the use of biological material and to the access to medical records.

## 5. Conclusions

High-sensitivity FCM-MRD demonstrated performance comparable to PCR-MRD, highlighting its utility as a reliable and complementary tool for MRD assessment in B-cell acute leukemia. Additionally, the immunophenotypic strategy employed showed a high specificity, with lymphoblastic cells consistently expressing established leukemia-associated markers and a distinctive CD34++CD38−/dim expression profile. This pattern may reflect a characteristic abnormal phenotype within this high-risk subgroup, providing valuable insights into the biology and clinical behavior of Philadelphia chromosome-positive B-cell acute leukemia. Further research, involving larger cohorts and standardized protocols, is necessary to validate and expand upon these results.

## Figures and Tables

**Table 1:** Immunophenotypic differences between Ph+ ALL cases and other B-ALL.

LAIP Expression	Ph+ ALL (*n* = 17)	Other B-ALL (*n* = 58)	*p*-Value *
CD66c+	14/20 tested ** (70.0%)	49 (84.5%)	0.755
CD73+	14 (82.3%)	47 (81.0%)	1.000
CD304+	15 (88.2%)	38 (65.5%)	0.026
CD73+/CD304+	13 (76.5%)	9 (15.5%)	<0.0001
CD66+/CD304+	10 (58.8%)	4 (6.90%)	<0.0001
CD66+/CD73+/CD304+	8 (47.0%)	25 (43.1%)	0.660
	**Ph+ ALL (*n* = 27)**	**Other B-ALL (*n* = 79)**	*p*-Value *
CD123+	19 (70.4%)	39 (49.4%)	0.074
CD10+	23 (85.2%)	62 (78.5%)	0.581
CD34+	26 (96.3%)	37 (46.8%)	<0.0001
CD38+	6 (22.2%)	46 (58.2%)	0.0016
CD34+/CD38 neg/dim	21 (77.8%)	21 (26.6%)	<0.0001

Legend: LAIP, leukemia-associated immunophenotype; Dim, low expression; Neg, negative expression; Ph+, Philadelphia chromosome; * Fisher exact test considering tested cases for each marker *n*(%). ** The marker CD66c was tested in 20 cases from the Ph+ ALL group.

**Table 2:** Revision of discrepant cases between FCM-MRD and PCR-MRD.

LOD FCM-MRD.	FCM-MRD ^1^	*BCR::ABL1*/ABL Ratio
<0.01%	Not detected	0.0070%
<0.01%	Not detected	0.0120%
<0.01%	Not detected	0.0400%
<0.01%	Not detected	0.0260%
<0.01%	Not detected	0.0320%
<0.01%	Not detected	0.0520%
<0.01%	Not detected	0.0520%
<0.01%	Not detected	0.1050%
<0.0002%	Not detected ^2^	Detected above 0.0001% ^2^
<0.0002%	Not detected ^2^	Detected above 0.0001% ^2^
<0.0002%	Not detected ^3^	Detected above 0.0001% ^3^
<0.001%	0.07% ^4^	Not detected

Legend: LoD Lower limit of detection. ^1^—Samples with low number of acquired events in FCM-MRD; ^2^—Samples with a transcript e14a2 detected but not quantified above LoD (<0.0001%); ^3^—Sample with e14a2 detected but not quantified and e1a2 not detected; ^4^—False positive FCM-MRD.

## Data Availability

Data is contained within the article and Supplementary Materials.

## Supplementary Materials

The following supporting information can be downloaded at: https://www.mdpi.com/article/10.3390/ijms26052116/s1.

## Abbreviations

ALL	Acute lymphoblastic leukemia
AYA	Adolescents and young adults
APS	Automatic population separation
B-ALL	B-lymphoblastic leukemia
CHC-UFPR	Complexo Hospital de Clínicas da Universidade Federal do Paraná
CML	Chronic myeloid leukemia
CML-BP	Chronic myeloid leukemia Blast Phase
DifN	Different from normal
FCM	Flow cytometry
FCM-MRD	Flow cytometry mensurable residual disease
ICC	International Consensus Classification
Ig/TCR	Immunoglobulin/TCR rearrangement
IHC	Immunohistochemistry
LAIP	Leukemia-associated immunophenotype
LCS	Leukemic Stem Cell
MRD	Mensurable residual disease
MPAL	Mixed phenotype acute leukemia
NGS	Next-generation sequencing
PCR-MRD	Polymerase chain reaction mensurable residual disease
Ph+ ALL	Philadelphia chromosome positive acute lymphoblastic leukemia
RQ-PCR	real-time quantitative polymerase chain reaction
SCT	Stem cell transplantation
T-ALL	T-lymphoblastic leukemia
TKIs	Tyrosine kinase inhibitors
