# Supplementary material for: Comprehensive Analysis of High-Sensitive Flow Cytometry and Molecular Mensurable Residual Disease in Philadelphia Chromosome-Positive Acute Leukemia
Source: Int J Mol Sci. 2025 Feb 27;26(5):2116. doi: 10.3390/ijms26052116 (PMC11900146; doi:10.3390/ijms26052116)
Supplement: Supplementary file 1 [file ijms-26-02116-s001.zip › Suplementary Table S1 S2 S3.pdf]

Supplementary Table S1: Patient Genetic-Molecular Characteristics.

Case n.	Classification	Age	Gender	*BCR::ALB transcript*	Karyotype
1	Ph+ ALL	3,25	M	*p190(e1a2)*	47~48,XY,t(9;22)(q34;q11.2)[13]
2	Ph+ ALL	3,80	F	*p190(e1a2)*	46~49,XX,del(2)(q22),+14,+16,+19,+22,+mar[cp11]/46,XX[15]
3	Ph+ ALL	4,00	M	*p190(e1a2)*	no metaphases
4	Ph+ ALL	4,80	M	*p190(e1a2)*	46,XY,?t(9;22)(q34;q11.2)[4]/46,sl,der(19)t(1;19)
5	Ph+ ALL	20,60	F	*p190(e1a2)*	46,XX,t(9;22)(q34;q11.2)[20]
6	Ph+ ALL	22,70	F	*p190(e1a2)*	46,XX,t(9;22)(q34;q11.2)[18]
7	Ph+ ALL	23,40	M	*p190(e1a2)*	46,XY,t(9;22)(q34;q11.2)[20]
8	Ph+ ALL	26,01	F	*p190(e1a2)*	46,XX,t(9;22)(q34;q11.2)[20]
9	Ph+ MPAL B/My	29,10	F	*p190(e1a2)*	39~44,X,-X,t(7;14)(p15;q11),t(9;22)(q34;q11.2),+16[20]
10	Ph+ ALL	30,70	F	*p190(e1a2)*	no metaphases
11	Ph+ ALL	41,40	M	*p190(e1a2)*	46,XY,der(14)t(8;14)(q11;q32),+mar,inc[3]/46,XY[17]
12	Ph+ ALL	42,90	M	*p190(e1a2)*	46,XY[20]
13	Ph+ ALL	47,10	F	*p190(e1a2)*	46,XX,t(9;22)(q34;q11.2)[15]
14	Ph+ ALL	49,00	F	*p190(e1a2)*	46 XX, del(10)(p?12), del 22(q11.2)[8], 46 XX [11]
15	Ph+ ALL	51,60	M	*p190(e1a2)*	52~56,XY...[6]/46,XY[11]
16	Ph+ ALL	64,20	F	*p190(e1a2)*	46,XY,t(9;22)(q34;q11.2)[17]
17	Ph+ ALL	69,80	M	*p190(e1a2)*	46,XY,t(9;22)(q34;q11.2)[12]
18	CML-BP	4,01	M	*p210 - e13a2(b2a2) / p190 (e1a2)*	46,XY,t(9;22)(q34;q11.2),+der(22)t(9;22)[16]/46,XY[4]
19	CML-BP	11,80	F	*p210 - e13a2(b2a2) / e14a2(b3a2) / p190 (e1a2)*	46,XX,t(9;22)(q34;q11.2)[20]
20	CML-BP	14,30	M	*p210 - e13a2(b2a2) / p190 (e1a2)*	46,XY,t(9;22)(q34;q11.2)[19]
21	CML-BP	31,90	F	*p210 - e13a2(b2a2) / e14a2(b3a2) / p190 (e1a2)*	46,XX,t(9;22)(q34;q11.2)[5]/46,XX[5]
22	CML-BP	36,60	M	*p210 - e13a2(b2a2) / p190 (e1a2)*	46,XY,t(9;22)(q34;q11.2)[20]
23	CML-BP	48,50	F	*p210 - e14a2(b3a2) / p190 (e1a2)*	46,XX[13]
24	CML-BP	52,60	M	*p210 - e14a2(b3a2) / p190 (e1a2)*	46,XY[20]
25	CML-BP	57,70	F	*p210 - e13a2(b2a2)*	46,XX,t(9;22)(q34;q11.2)[16]
26	CML-BP	63,00	M	*p210 - e13a2(b2a2)*	46,XY,t(9;22)(q34;q11.2)[12]
27	CML-BP	75,60	M	*p210 - e14a2(b2a2)*	46,XY,t(9;22)(q34;q11.2)[20]

Supplementary Table S2: Patient Clinic Characteristics

Case n	Classification2	ALL subtype	Clinic characteristic	Hb (g/L)	WBC (×10⁹/L)	Platlets (×10⁹/L)	HSCT	Relapse	Outcome
1	Ph+ ALL	B-Common ALL	D15 positivity (>5%), CNS infiltration	7.10	3.81	26	0	0	0
2	Ph+ ALL	B-Common ALL	Ph+ ALL	6.40	343	578	1	0	0
3	Ph+ ALL	Pro-B ALL	BCP-ALL late relapse	8.50	23.0	46.6	1	1	1
4	Ph+ ALL	B-Common ALL	Ph+ ALL / t(1;19)	9.30	10.6	52	0	0	0
5	Ph+ ALL	B-Common ALL	Extramedular breast infiltration				1	1	0
6	Ph+ ALL	B-Common ALL	Ph+ ALL				1	0	0
7	Ph+ ALL	B-Common ALL	Ph+ ALL	10.7	97.5	174	1	1	0
8	Ph+ ALL	B-Common ALL	Ph+ ALL	8.00	2.84	322	1	0	0
9	Ph+ MPAL B/My	MPAL B/My	MPAL/Extramedular liver infiltration	8.30	4.51	9	1	0	0
10	Ph+ ALL	B-Common ALL	Ph+ ALL	9.30	5.52	7	1	0	1
11	Ph+ ALL	B-Common ALL	Ph+ ALL / t(8;14)	6.20	47.26	17	1	0	0
12	Ph+ ALL	Pro-B ALL	Ph+ ALL	5.20	4.32		1	0	1
13	Ph+ ALL	Pro-B ALL	BCP-ALL late relapse				1	1	1
14	Ph+ ALL	B-Common ALL	Ph+ ALL	10.0	5.73	8	1	0	0
15	Ph+ ALL	B-Common ALL	High D15 positivity (>5%)	9.60	5.68	6	0	0	1
16	Ph+ ALL	B-Common ALL	High D15 positivity (>5%)	7.90	3.99	11.6	0	1	1
17	Ph+ ALL	B-Common ALL	High D15 positivity (>5%)	2.7	36.9	10	0	0	1
18	CML-BP	CML-BP B-Common	High WBC	3,4	498.0	121	1	0	0
19	CML-BP	CML-BP B-Common	CNS infiltration	12.80	4.73	246	0	1	0
20	CML-BP	CML-BP B-Common	High WBC with neutrophilia	10.0	221.8	105	1	0	0
21	CML-BP	CML-BP B-Common	High WBC	6.20	76.1	37	1	0	1
22	CML-BP	CML-BP B-Common	Previous CML history, TKI use	7.90	12.11	21.9	1	0	1
23	CML-BP	CML-BP B-Common	High D15 positivity (>5%)	5.00	3.5	10.7	1	0	0
24	CML-BP	CML-BP Pro-B	Previous CML history, TKI use				1	1	1
25	CML-BP	CML-BP B-Common	High WBC	8.0	39.6	17	1	0	1
26	CML-BP	CML-BP B-Common	High WBC with neutrophilia				0	1	0
27	CML-BP	CML-BP B-Common	High WBC with neutrophilia	11,1	181.5	43	0	0	1

Supplementary Table S3: Immunophenotipic Characteristics

Case n	Maturation	LAIP markers	CD10	CD34	CD38	CD66C	CD73	CD304	CD123	D15 2	d15	d33	Sem 12
1	CD34+CD38-	CD34+CD38-	(+)	(++)	(-)				(+)	D15 >5%	35.4	0.0000	0.0000
2	CD34+CD38-	CD66+CD73+CD304+	(+)	(++)	(-/+)	(-/++)	(+)	(+)	(+)	D15 pos	0.25	0.0000	0.0000
3	CD10-CD34+CD38+	CD73+	(-/+ NG2)	(+)	(++)	(-)	(+)	(-)	(-/+)	D15 >5%	na	na	na
4	CD34+CD38-	CD73+CD304+	(+)	(-)	(++)	(-)	(++)	(+)	(+)	D15 pos	0.52	0.0000	0.0000
5	CD34+CD38-	CD66+	(+)	(+)	(-)	(+)	(-)	(-)	(-)	D15 >5%	50.0	0.0000	0.0000
6	CD34+CD38-	CD34+CD38-	(+)	(++)	(-)				(+)	D15 >5%	na	na	na
7	CD34+CD38-	CD34+CD38-	(+)	(++)	(-)				(+)	D15 >5%	na	na	na
8	CD34+CD38-	CD34+CD38-	(+)	(++)	(-)				(+)	D15 >5%	na	na	na
9	CD34+CD38-	CD66+CD73+CD304+	(+)	(++)	(-)	(-/++)	(+)	(++)	(-/+)	D15 >5%	29.4	7.20	0.0600
10	CD34+CD38-	CD66+CD73+CD304+	(+)	(++)	(-)	(-/++)	(++)	(++)	(+)	D15 pos	1.95	0.80	0.6000
11	CD34+CD38-	CD34+CD38-	(+)	(++)	(-/+)	CD9++			(+)	D15 <0.01%	0.00	0.0000	0.0000
12	CD10-CD34+CD38+	CD66+CD73+CD304+	(-)	(++)	(++)	(-/++)	(+)	(+)	(-/+)	D15 pos	0.18	0.0037	0.0000
13	CD10-CD34+CD38+	CD73+CD304+	(-)	(+)	(++)	(-)	(++)	(++)	(-/+)	D15 pos	1.10	0.2100	na
14	CD34+CD38-	CD66+	(+)	(++)	(-)	(-/++)			(+)	D15 pos	1.90	0.0000	0.0000
15	CD34+CD38-	CD73+CD304+	(+)	(+)	(++)	(-)	(++)	(++)	(-/+)	D15 >5%	17.0	43.5	death
16	CD34+CD38-	CD73+CD304+	(+)	(++)	(-)	(-)	(+)	(+)	(+)	D15 >5%	7.40	0.070	0.0014
17	CD34+CD38-	CD66+	(-/++)	(+)	(-/+)	(+)			(+)	D15 >5%	80.0	death	death
18	CD34+CD38-	CD66+CD73+CD304+	(+)	(++)	(-)	(-/++)	(++)	(+)	(+)	D15 >5%	15.90	0.0300	0.0000
19	CD34+CD38-	CD34+CD38-	(+)	(++)	(-)				(+)	D15 >5%	na	na	na
20	CD34+CD38-	CD34+CD38-	(+)	(++)	(-)				(+)	D15 <0.01%	0.00	0.0000	0.0000
21	CD34+CD38-	CD66+CD73+CD304+	(+)	(+)	(-)	(-/++)	(+)	(+)	(+)	D15 pos	0.03	0.0015	0.0000
22	CD34+CD38-	CD66+CD304++	(-/++)	(++)	(-)	(-/++)	(-)	(+)	(+)	D15 pos	0.07	0.0000	0.0000
23	CD34+CD38-	CD73+CD304+	(+)	(++)	(-)	(-)	(+)	(+)	(+)	D15 >5%	63.00	28.0	0.0000
24	CD10-CD34+CD38+	CD66+CD304++	(-)	(+)	(++)	(-/++)	(-)	(++)	(-/+)	D15 pos	0.83	88.4	death
25	CD34+CD38-	CD66+	(+)	(++)	(-)	(-/++)			(+)	D15 >5%	na	0.0000	0.0000
26	CD34+CD38-	CD66+CD73+CD304+	(+)	(++)	(-)	(-/++)	(+)	(+)	(-/+)	D15 >5%	65.00	11.9	7.5000
27	CD34+CD38-	CD66+CD73+CD304+	(+)	(+)	(-/+)	(-/++)	(+)	(+)	(+)	D15 pos	2.52	death	death
