# Supplementary material for: Comprehensive Analysis of High-Sensitive Flow Cytometry and Molecular Mensurable Residual Disease in Philadelphia Chromosome-Positive Acute Leukemia
Source: Int J Mol Sci. 2025 Feb 27;26(5):2116. doi: 10.3390/ijms26052116 (PMC11900146; doi:10.3390/ijms26052116)
Supplement: Supplementary file 1 [file ijms-26-02116-s001.zip › Suplementary Table S4.pdf]

Supplementary Table S4: FCM and PCR values and concordance

Sample	FCM (% blasts)	PCR (transcript level)	CONCORDANCE
1	0.0	0.0	CONCORDANT
2	0.0	0.0	CONCORDANT
3	0.0	0.0	CONCORDANT
4	0.0	0.0	CONCORDANT
5	0.0	0.0	CONCORDANT
6	0.0	0.0	CONCORDANT
7	0.0	0.0	CONCORDANT
8	0.0	0.0	CONCORDANT
9	0.0	0.0	CONCORDANT
10	0.0	0.0	CONCORDANT
11	0.0	0.0	CONCORDANT
12	0.0	0.0	CONCORDANT
13	0.0	0.0	CONCORDANT
14	0.0	0.0	CONCORDANT
15	0.0	0.0	CONCORDANT
16	0.0	0.0	CONCORDANT
17	0.0	0.0	CONCORDANT
18	0.0	0.0	CONCORDANT
19	0.0	0.0	CONCORDANT
20	0.0	0.0	CONCORDANT
21	0.0	0.0	CONCORDANT
22	0.0	0.0	CONCORDANT
23	0.0	0.0	CONCORDANT
24	0.0	0.0	CONCORDANT
25	0.0	0.0	CONCORDANT
26	0.0	0.0	CONCORDANT
27	0.0	0.0	CONCORDANT
28	0.0	0.0	CONCORDANT
29	0.0	0.0	CONCORDANT
30	0.0	0.0	CONCORDANT
31	0.0	0.0	CONCORDANT
32	0.0	0.0	CONCORDANT
33	0.0	0.0	CONCORDANT
34	0.0	0.0	CONCORDANT
35	0.0	0.0	CONCORDANT
36	0.0	0.0	CONCORDANT
37	0.0	0.0	CONCORDANT
38	0.0	0.0	CONCORDANT
39	0.0	0.0	CONCORDANT
40	0.0	0.0	CONCORDANT
41	0.0	0.0	CONCORDANT
42	0.0	0.0	CONCORDANT
43	0.0	0.0	CONCORDANT
44	0.0	0.0	CONCORDANT
45	0.0	0.0	CONCORDANT
46	0.0	0.0	CONCORDANT
47	0.0	0.0	CONCORDANT
48	0.0	0.0	CONCORDANT
49	0.0	0.0	CONCORDANT
50	0.0	0.0	CONCORDANT
51	0.0	0.0	CONCORDANT
52	0.0	0.105	DISCORDANCE low FCM events
53	0.0	0.052	DISCORDANCE low FCM events
54	0.0	0.052	DISCORDANCE low FCM events
55	0.0	0.04	DISCORDANCE low FCM events
56	0.0	0.032	DISCORDANCE low FCM events
57	0.0	0.026	DISCORDANCE low FCM events
58	0.0	0.017	DISCORDANCE low FCM events
59	0.0	0.012	DISCORDANCE low FCM events
60	0.0	0.007	DISCORDANCE low FCM events
61	0.0	0.0001	DISCORDANCE PCR detected below QR
62	0.0	0.0001	DISCORDANCE PCR detected below QR
63	0.0	0.0001	DISCORDANCE PCR detected below QR**
64	0.0007	0.002	CONCORDANT
65	0.0017	0.0001	CONCORDANT
66	0.002	0.003	CONCORDANT
67	0.002	0.0001	CONCORDANT
68	0.003	0.0001	CONCORDANT
69	0.0032	0.0001	CONCORDANT
70	0.004	0.067	CONCORDANT
71	0.0045	0.085	CONCORDANT
72	0.013	0.016	CONCORDANT
73	0.0155	0.017	CONCORDANT
74	0.02	1.042	CONCORDANT
75	0.03	0.137	CONCORDANT
76	0.06	0.012	CONCORDANT
77	0.07	22.1	CONCORDANT
78	0.07	0.0	DISCORDANCE; MFC false pos
79	0.39	0.106	CONCORDANT
80	0.83	0.2	CONCORDANT
81	1.0	0.832	CONCORDANT
82	1.0	1.261	CONCORDANT
83	1.25	4.73	CONCORDANT
84	1.87	0.015	CONCORDANT
85	1.87	1.248	CONCORDANT
86	100.0	173.0	CONCORDANT
87	18.0	0.844	CONCORDANT
88	18.0	0.51	CONCORDANT
89	21.0	20.0	CONCORDANT
90	26.4	20.0	CONCORDANT
91	28.0	1.261	CONCORDANT 2 LOGS
92	4.5	0.027	CONCORDANT
93	43.7	60.49	CONCORDANT
94	57.0	0.033	CONCORDANT 2 LOGS
95	63.0	1.261	CONCORDANT
96	7.3	26.04	CONCORDANT
97	72.0	50.0	CONCORDANT
98	75.0	51.0	CONCORDANT
99	81.0	138.0	CONCORDANT
100	82.0	100.0	CONCORDANT
101	84.0	0.053	CONCORDANT 2 LOGS
102	87.7	0.012	CONCORDANT 2 LOGS
103	88.0	100.0	CONCORDANT
104	9.8	6.272	CONCORDANT
105	90.0	90.0	CONCORDANT
106	90.0	90.0	CONCORDANT
107	90.0	116.0	CONCORDANT
108	92.0	80.0	CONCORDANT
109	94.0	1.386	CONCORDANT 2 LOGS

Legend: FCM, flow cytometry; PCR, polymerase chain reaction; QR, quantitative range; **0.0007% of blasts detected after revision in FCM.
