# Supplementary material for: Comprehensive Analysis of High-Sensitive Flow Cytometry and Molecular Mensurable Residual Disease in Philadelphia Chromosome-Positive Acute Leukemia
Source: Int J Mol Sci. 2025 Feb 27;26(5):2116. doi: 10.3390/ijms26052116 (PMC11900146; doi:10.3390/ijms26052116)
Supplement: Supplementary file 1 [file ijms-26-02116-s001.zip › Suplementary Table S5 S6.pdf]

Table S5: Example of MRD kinetic from a CML-BP patient (case 23) with two BCR::ABL1 transcripts presented as acute leukaemia - p190 (e1a2) and p210 (e14a2) detected

Patient 23 -Time point	FCM-MRD %	Number of events in FCM	PCR-MRD	FCM vs PCR MRD
Diagnoses	43.7%	1,000,000	60.5%	C
After induction	63.0%	1,000,000	1.26% [e1a2 and e14a2 detected]	C
After consolidation	28.0%	1,000,000	Not done	--
Before SCT	0.0155%	10,000,000	0.017% [e1a2 and e14a2 detected]	C
D30	0.0007%**	10,000,000	0.0002% [e14a2 detected below QR]	D
D60	0.0017%	10,000,000	0.0001% [e14a2 detected below QR]	C
D100	<0.001%	10,000,000	<0.0001% [not detected]	C
D180	0.002%	10,000,000	0.0001% [e14a2 detected below QR]	C
D240	<0.0001%	10,000,000	<0.0001% [not detected]	C
D360	<0.001%	2,000,000	<0.0001% [not detected]	C

Legend: C, concordant results; D, discordant results; FCM-MRD, flow cytometry mensurable residual disease; LoD, limit of detection; SCT, hematopoietic stem cell transplantation; PCR, polymerase chain reaction; QR, quantitative range; **0.0007% of blasts detected after revision.

Table S6: Example of MRD kinetic from a CML-BP patient (case 23) with three BCR::ABL1 transcripts presented as acute leukaemia - p190 (e1a2) and p210 (E13a2 and e14a2) detected

Patient 21 - Time point	FCM-MRD %	Number of events in FCM	PCR-MRD	FCM vs PCR MRD
Diagnoses	80.0%	1,000,000	110%	C
1. Hyper-CVAD +TKI	0.031%	10,000,000	0.03% [e1a2 / e13a2 and e14a2 detected]	C
2. Hyper-CVAD+TKI	0.0015%	10,000,000	0.003% [e13a2 and e14a2 detected]	C
3. Hyper-CVAD+TKI	<0.001%	10,000,000	<0.0001% [not detected]	C
4. Hyper-CVAD+TKI	<0.001%	10,000,000	<0.0001% [not detected]	C
5. Hyper-CVAD+TKI	<0.001%	10,000,000	<0.0001% [not detected]	C
6. Hyper-CVAD+TKI	<0.001%	10,000,000	<0.0001% [not detected]	C

Legend: C, concordant results; D, discordant results; FCM-MRD, flow cytometry mensurable residual disease; LoD, limit of detection; ; PCR, polymerase chain reaction; QR, quantitative range; TKI, tyrosine kinase inhibitor.
